# A unique case of a fulminant clonal CD8-positive T-cell lymphoproliferative disorder with CNS involvement

**DOI:** 10.1186/s12883-023-03371-8

**Published:** 2023-09-15

**Authors:** Lia Mesbah-Oskui, Jarrah Alabkal, Waleed Alduaij, Priya S. Dhawan

**Affiliations:** 1 Division of Neurology, Faculty of Medicine, University of British Columbia, Vancouver, BC Canada; 2 Division of Medical Oncology, Faculty of Medicine, University of British Columbia, Vancouver, BC Canada; 3 Centre for Lymphoid Cancer, British Columbia Cancer, Vancouver, BC Canada

**Keywords:** CNS lymphoma, Primary CNS lymphoma, T-cell lymphoproliferative disorder

## Abstract

**Background:**

This is a unique case that describes the presentation, investigations, and disease trajectory of a fatal, clonal CD8-positive T-cell lymphoproliferative disorder in an otherwise healthy and immunocompetent patient with Epstein-Barr virus seronegative status. Central nervous system involving T-cell lymphoproliferative disorders are rare and typically encountered in the setting of immunocompromise. These disorders are often associated with aggressive cytomorphological features and characteristic magnetic resonance imaging patterns, which were not seen in this case.

**Case presentation:**

Here we describe a case of a 65 year-old male presenting with neuropsychiatric symptoms, truncal ataxia, and falls who’s bone marrow, cerebrospinal fluid, and brain biopsy were consistent with a clonal CD8-positive T-cell lymphoproliferative disorder that did not meet existing World Health Organization criteria for classification as T-cell lymphoma. The patient was treated with intrathecal methotrexate resulting in transient improvement of his symptoms followed by disease progression and death related to aspiration.

**Conclusions:**

This case highlights the importance of urgent and comprehensive work-up in patients with clinical features suggestive of lymphoma with central nervous system involvement, despite atypical imaging features and lack of cytomorphological features satisfying current World Health Organization classification criteria.

## Background

Unclassifiable T-cell lymphoproliferative disorders (LPD) are a rare and heterogeneous group of poorly characterized disorders associated with clonal T-cell proliferation that do not fulfill the diagnostic criteria for known T-cell lymphoma/leukemia entities [1]. Central nervous system (CNS) involvement by such T-cell LPDs is even rarer, with an unknown incidence and prevalence and unclear disease trajectory. This is not surprising given the overall uncommon nature of T-cell lymphomas, which comprise less than 15% of non-Hodgkin lymphomas. CNS involvement in T-cell lymphoma is estimated to occur in about 2% of cases and is associated with a poor prognosis characterized by an overall survival of less than 2 years [1, 2]. Similarly, T-cell CNS lymphoma comprises less than 4% of total primary CNS lymphoma (PCNSL) cases in North America [3]. Here we describe a case of a clonal CD8-positive T-cell LPD with CNS involvement in a previously healthy patient with isolated neurologic symptoms. Although the CD8-positive T-cell LPD had no cytomorphological features of T-cell lymphoma, it was associated with a dismal outcome, despite the use of a CNS-penetrating lymphoma chemotherapy regimen. To our knowledge, this is the first report that provides insight into the presentation, laboratory features, and disease trajectory of a fatal, clonal CD8-positive T-cell LPD in an otherwise healthy, immunocompetent patient with Epstein-Barr virus (EBV) negative serostatus.

## Case presentation

A previously healthy 65-year-old male presented with a 3-month history of fatigue and frequent unexplained falls. This occurred in the context of an approximate 1.5-year history of progressive cognitive decline, leading to dependence on his wife for instrumental activities of daily living, and behavioral changes. The patient’s wife reported that he had grown withdrawn and less engaged in social settings and had been in 3 motor vehicle accidents over the past year, resulting in the suspension of his license.

On examination he had reduced verbal output, but was otherwise alert, attentive, and oriented to place and person. Attention and language were normal. He was able to obey complex, multi-step commands. He was noted to have mildly restricted upgaze. The remainder of his cranial nerve examination was unremarkable. The patient had severe truncal ataxia, such that he was unable to maintain an upright posture even while seated and was unable to stand or walk. There were no features of appendicular ataxia or tremor. His tone was normal throughout. His patellar reflexes were brisk with the presence of crossed adductor responses. He had bilateral flexor plantar responses. Sensory and motor examination were otherwise normal. He was not safe to mobilize for gait assessment.

**Fig. 1:**
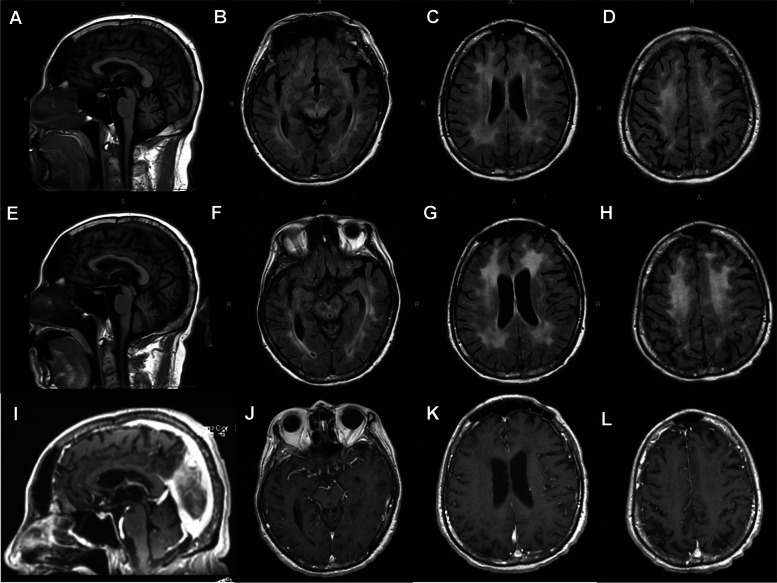
MRI Brain. T1-weighted sagittal without contrast (**A**, **E**), T2 FLAIR coronal (**B**-**D**, **F**–**H**), MPR of T1-weighted post-gadolinium sagittal (**I**) and T1- weighted post-gadolinium coronal (**J**-**L**) sections showing diffuse periventricular and deep white matter hyper-intensities with interval progression over 4 months (**A**-**D** vs. **E**-**L**). MRI: magnetic resonance imaging, FLAIR: fluid-attenuated inversion recovery, MPR: multiplanar reformation

Computed tomography (CT) of the head was notable for a depressed fracture of the left frontal sinus explained by a recent fall. Vascular imaging and magnetic resonance imaging (MRI) of the cervical spine were unremarkable. MRI of the brain, however, identified extensive, confluent T2 hyperintensities in the subcortical and deep white matter with diffuse hemispheric involvement and extension into the brainstem and cerebellum (Fig. 1 A-D). CT neck/chest/abdomen/pelvis showed no lymphadenopathy. Positron emission tomography (PET) scan showed no hypermetabolic lesions to suggest malignancy.

Additional investigations included lumbar puncture and serology testing. Blood work was largely unremarkable with normal B12, TSH, vitamin E, and serum/urine protein electrophoresis. The rheumatologic panel showed ANA, PR3, MPO and rheumatoid factors that were all negative. C-reactive protein was mildly elevated at 4.4 mg/L (normal < 3.1 mg/L). Infectious workup was negative for syphilis, HIV and hepatitis C. Hepatitis B core antibody was positive. JC virus and HHV-6 testing were not performed. Serum lactate dehydrogenase level was normal. Lumbar puncture was notable for a lymphocytic pleocytosis with an associated elevation in protein (WBC 36 cells/µL and protein 0.85 g/L; reference range 0–5 cells/µL and 0.15–0.45 g/L, respectively). Infectious and paraneoplastic antibody panel studies of cerebrospinal fluid (CSF) and serum were unremarkable. CSF was sent for flow cytometry, which revealed an abnormal clonal CD8 T-cell population. Bone marrow biopsy showed involvement by the same abnormal CD8-positive T-cell population in a nodular and interstitial pattern occupying approximately 20% of the cellular marrow, consistent with a diagnosis of a CD8-positive T-cell LPD that is not further classifiable. Multiplex polymerase chain reaction T-cell clonality studies using TCRB BIOMED-2 primer sets identified the same clonal T-cell population in the CSF and bone marrow [4]. Lymphoma tumor board review concluded that the MRI changes were not characteristic of T-cell lymphoma. Moreover, given that transient clonal T-cell populations can occur in the context of infections and inflammatory diseases, the identified clonal CD8-positive T-cell population was favored to be an epiphenomenon of a yet undiagnosed infectious/inflammatory process rather than the major driver of the patient’s clinical presentation [5]. Therefore, further neurologic work-up for alternative etiologies was recommended.

**Fig. 2:**
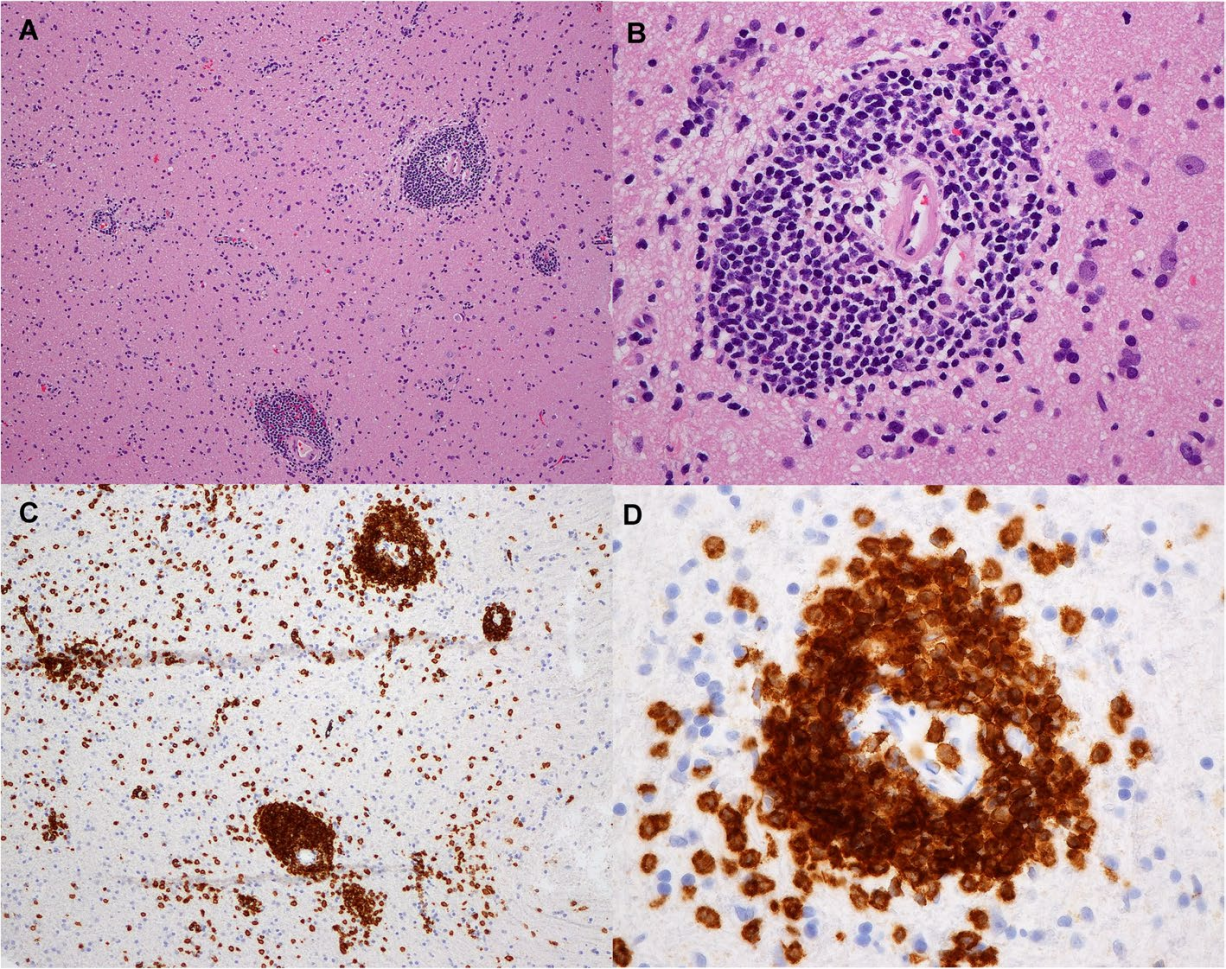
Histologic sections from brain biopsy. Hematoxylin and eosin stains at 10 (**A**) and 40 (**B**) magnification reveal fragments of brain tissue containing a sparse perivascular lymphocytic infiltrate composed of small cells with mildly irregular nuclei, mature chromatin, inconspicuous nucleoli, and scant cytoplasm. There is no overt tissue destruction or necrosis, and mitotic figures are not identified. CD8 immunostain at 10 (**C**) and 40 (**D**) magnification show that perivascular lymphocytes are CD8 positive. Additional immunohistochemical studies confirmed the T-cell phenotype (not shown)

The patient re-presented one month later to the hospital following an aspiration event. A repeat MRI of the brain four months after his initial scan, showed progression of the diffuse white matter changes with extensive changes in the midbrain (Fig. 1E-L). Repeat metabolic, autoimmune, and infectious workup remained unremarkable. Repeat bone marrow biopsy was not performed at this time. The patient received a course of antimicrobial therapy for his aspiration event with no improvement in his mental status. Steroid pulse was pursued, given his negative infectious workup and persistent neurologic symptoms. He exhibited significant improvement in his level of consciousness after he received a 5-day pulse of 1 mg/kg methylprednisolone. He was then maintained on 50 mg of prednisone daily. A right frontal brain biopsy was performed and pathology identified an angiocentric T-cell infiltrate involving parenchymal small blood vessels, with atypical CD8-positive T-lymphocytes within the perivascular and intramural space (Fig. 2 A-D). Additional immunohistochemical studies showed that these lymphocytes were positive for TCR-beta-F1, TIA-1 and CD57 (minor subset), but negative for CD4 and CD56. Interestingly, the lymphocyte population showed no aggressive cytomorphological features and a Ki67 proliferative index of virtually 0% (Fig. 2 A-B). In situ hybridization for EBV RNA (EBER) was negative. T-cell clonality studies identified a clonal CD8-positive T-cell population identical to that identified in the bone marrow and CSF.

The patient’s presentation, cytomorphological and molecular analysis of diagnostic material are consistent with a clonal CD8-positive T-cell LPD with CNS involvement that did not meet any of the WHO classification’s diagnostic categories for lymphoid neoplasms [1]. A diagnosis of lymphoma could not be rendered due to the bland T-cell cytomorphology, very low/absent proliferation and lack of destructive features. Although the WHO diagnostic entity of peripheral T-cell lymphoma, not otherwise specified, allows for indolent small CD8-positive T-cells, destructive lesions are required for the diagnosis. EBER negativity ruled out the possibility of EBV-positive LPDs. The lack of additional masses or lymphadenopathy on imaging and the identification of the same clonal CD8-positive T-cell LPD in all sampled sites of disease (brain, CSF and bone marrow) indicates that this clonal CD8-positive T-cell proliferation was indeed the predominant pathological feature.

After receiving one cycle of CHOP chemotherapy, treatment of the CNS disease was prioritized. CNS-directed therapy with CNS-penetrating chemotherapy, typically used for aggressive CNS lymphoma, was initiated and included 3 cycles of high-dose methotrexate with temozolomide added on the second and third cycles. Initially there was some improvement in the patient’s mental status; however, he remained very unsteady and unable to perform a number of activities of daily living, necessitating long-term care. Unfortunately, the patient experienced recurrent aspiration events and was transitioned to palliative care. He died approximately 1 year and one month following medical presentation and 2.5 years after symptom onset.

## Discussion

Here we describe a unique case of an immunocompetent, EBV-negative, patient with fatal CD8-positive T-cell LPD characterized by infiltration of the bone marrow, CSF, and brain parenchyma. T-cell LPDs with CNS involvement are considered extremely rare and reports are limited to immunosuppressed populations, particularly in the post-transplant setting where EBV-driven B-cell LPDs remain much more common [2, 6]. To our knowledge, only one other case of a fatal T-cell LPD was previously reported [6]. However, this occurred in the context of treatment of multiple sclerosis with the monoclonal anti-CD52 antibody alemtuzumab, which is associated with significant immune suppression due to profound B- and T-cell depletion [7].

Little is known about T-cell LPDs involving the CNS, but some clinical features overlap with those of T-cell PCNSL (PCNSTL). Like PCNSTL, our case shows a typical presentation with non-specific behavioral, cognitive, and neuropsychiatric changes in a characteristic demographic [8]. In contrast to PCNSL, where MRI typically reveals a solitary contrast-enhancing lesion in 60–70% of cases, our case demonstrated confluent T2 hyperintensities [9]. This highlights the variable radiographic presentation of CNS T-cell LPDs and illustrates the importance of investigating patients with otherwise unexplained, confluent T2 hyperintensities, even in the absence of typical features, such as gadolinium enhancement. It is important to recognize that these lesions can be associated with an aggressive disease course even in the absence of bone fide lymphoma diagnosis, prompting urgent diagnostic and therapeutic interventions.

Blood–brain-barrier penetrating doses of methotrexate (more than 1.5 g/m^2^ intravenous) form the cornerstone of PCNSL management, and was part of our patient’s care [3]. This treatment resulted in transient improvement in our patient’s mental status, but did not significantly impact disease progression. In conclusion, we report a rare case of a clonal CD8-positive T-cell LPD with CNS infiltration culminating in rapid neurologic decline and death without cytomorphological features of lymphoma/malignancy. Our case illustrates the need for urgent comprehensive work-up in patients with typical clinical features of PCNSL despite atypical imaging and cytomorphological features, in order to provide timely intervention and counseling. Further studies are required to inform the optimal management of these very rare LPDs.

## Data Availability

Not applicable.

## Abbreviations

LPD: Lymphoproliferative disorders
WHO: World Health Organization
CNS: Central nervous system
PCNSL: Primary CNS lymphoma
EBVE: Pstein-Barr virus
CT: Computed tomography
MRI: Magnetic resonance imaging
PET: Positron emission tomography
CSF: Cerebrospinal fluid
EBER: In situ hybridization for EBV RNA
PCNSTL: T-cell PCNSL
